# Considerations for the Design and Implementation of COVID-19 Contact Tracing Apps: Scoping Review

**DOI:** 10.2196/27102

**Published:** 2021-06-09

**Authors:** Esli Osmanlliu, Edmond Rafie, Sylvain Bédard, Jesseca Paquette, Genevieve Gore, Marie-Pascale Pomey

**Affiliations:** 1 Research Institute of the McGill University Health Centre Montreal, QC Canada; 2 Division of Pediatric Emergency Medicine, Department of Pediatrics Montreal Children’s Hospital McGill University Health Centre Montreal, QC Canada; 3 Research Centre of the University of Montreal Hospital Centre Montreal, QC Canada; 4 Centre d’excellence sur le partenariat avec les patients et le public Montreal, QC Canada; 5 Schulich Library of Physical Sciences, Life Sciences, and Engineering McGill University Montreal, QC Canada; 6 Department of Health Policy, Management and Evaluation School of Public Health University of Montreal Montreal, QC Canada

**Keywords:** COVID-19, contact tracing, exposure notification, app, design, implementation, participatory, eHealth, surveillance, monitoring, review

## Abstract

**Background:**

Given the magnitude and speed of SARS-CoV-2 transmission, achieving timely and effective manual contact tracing has been a challenging task. Early in the pandemic, contact tracing apps generated substantial enthusiasm due to their potential for automating tracing and reducing transmission rates while enabling targeted confinement strategies. However, although surveys demonstrate public interest in using such apps, their actual uptake remains limited. Their social acceptability is challenged by issues around privacy, fairness, and effectiveness, among other concerns.

**Objective:**

This study aims to examine the extent to which design and implementation considerations for contact tracing apps are detailed in the available literature, focusing on aspects related to participatory and responsible eHealth innovation, and synthesize recommendations that support the development of successful COVID-19 contact tracing apps and related eHealth technologies.

**Methods:**

Searches were performed on five databases, and articles were selected based on eligibility criteria. Papers pertaining to the design, implementation, or acceptability of contact tracing apps were included. Articles published since 2019, written in English or French, and for which the full articles were available were considered eligible for analysis. To assess the scope of the knowledge found in the current literature, we used three complementary frameworks: (1) the Holistic Framework to Improve the Uptake and Impact of eHealth Technologies, (2) the Montreal model, and (3) the Responsible Innovation in Health Assessment Tool.

**Results:**

A total of 63 articles qualified for the final analysis. Less than half of the selected articles cited the need for a participatory process (n=25, 40%), which nonetheless was the most frequently referenced item of the Framework to Improve the Uptake and Impact of eHealth Technologies. Regarding the Montreal model, stakeholder consultation was the most frequently described level of engagement in the development of contact tracing apps (n=24, 38%), while collaboration and partnership were cited the least (n=2, 3%). As for the Responsible Innovation in Health framework, all the articles (n=63, 100%) addressed population health, whereas only 2% (n=1) covered environmental considerations.

**Conclusions:**

Most studies lacked fundamental aspects of eHealth development and implementation. Our results demonstrate that stakeholders of COVID-19 contact tracing apps lack important information to be able to critically appraise this eHealth innovation. This may have contributed to the modest uptake of contact tracing apps worldwide. We make evidence-informed recommendations regarding data management, communication, stakeholder engagement, user experience, and implementation strategies for the successful and responsible development of contact tracing apps.

## Introduction

### Background

As the global battle against the COVID-19 pandemic continues, the SARS-CoV-2 virus has infected over 160 million people and claimed over 3.3 million lives by May 2021 [1]. Even as vaccination programs expand their reach, nonpharmaceutical interventions such as social distancing, isolation, and quarantining remain essential for reducing viral transmission. Many will not have access to the vaccine until later in 2021, as limited production and delivery capacity warrants prioritizing high-risk groups. Many countries risk even longer delays in gaining access, despite efforts to promote a more equitable global distribution of the vaccine [2]. Moreover, long-term immunity from vaccination is not assured, and new virus variants have been associated with increased contagiosity [3]. Nonpharmaceutical interventions must therefore continue to support the fight against COVID-19, in complement to the global ramping up of vaccination.

Contact tracing is a fundamental containment strategy in response to emerging outbreaks. Public health agencies aim to rapidly identify individuals who may have been exposed to a person who is infected to recommend the most appropriate course of action (eg, self-isolation, symptom recording, and testing). The incubation period of this virus can last up to 14 days, during which infected individuals can unsuspectingly contaminate others [4,5]. Presymptomatic transmission along with other epidemiological, social, economic, and political challenges [6], coupled with chronic underfunding of public health systems [7], have undermined the reach of manual contact tracing during the COVID-19 pandemic.

### Contact Tracing Apps as eHealth Solutions During the COVID-19 Pandemic

A variety of eHealth solutions, which leverage information and communication technologies for the betterment of health and health care services [8], have been proposed in response to the pandemic [9]. Early in the pandemic, digital contact tracing rapidly emerged as a promising tool to support manual tracers [10,11] and enable a more selective approach to regional lockdowns [12]. Contact tracing apps constitute an example of eHealth aimed at supporting standard nonpharmaceutical interventions. Generally, the intended use is to digitally collect information within their network of users to reduce pathogen transmission. A recent review of international technological innovations developed in response to the pandemic listed almost 100 tracing applications at different stages of development, most of which were smartphone-based [13]. These can be further categorized as position tracking applications, which aim to enforce the quarantine of infected individuals, and the more commonly deployed contact tracing applications, which continuously measure distances between users to rapidly notify the high-risk contacts of an individual with a confirmed SARS-CoV-2 infection. The latter type predominates internationally, as it is more compatible with civil liberties. Although the term “contact tracing app” is typically used in academic texts and the general press, the primary purpose of such tools is to rapidly identify and notify individuals who have had a high-risk exposure [10]. Terminology matters as it may erode public trust in tools that are perceived to enable state surveillance of individual mobility (*tracing*, *tracking*). In this context, the contact tracing technology codeveloped by Apple and Google refers to an “Exposure Notification System” [14]. Similarly, the government of Canada encourages the public to use COVID Alert, an “exposure notification app,” which it describes as “an additional tool to protect yourself and your loved ones” [15].

Contact tracing apps can differ according to eight fundamental characteristics. First, their installation can be voluntary or compulsory. Second, the extent of informed consent varies between apps. Third, some apps use a decentralized data management strategy, while others enable linkages with governmental agencies. Fourth, their ability to detect contact between users can rely on technologies such as GPS, Bluetooth, or Quick Response codes. Fifth, the specific algorithms deployed in the back end of these apps will determine their output (eg, the calculation of a risk of infection or the tracing of potential contacts). Sixth, they require varying levels of human oversight, if at all present. Seventh, the degree of interaction with users regarding recommended actions (eg, testing and isolation recommendations) and the extent of interaction with public health agencies can differ. Last, safety protocols for data privacy may also vary [13,16,17]. Contact tracing apps thus refer to a heterogeneous cluster of eHealth tools that likely differ in effectiveness and uptake depending on their respective design characteristics.

### App Effectiveness, Barriers to Adoption, and Facilitators

Given their fundamental mechanism, the effectiveness of contact tracing apps depends in part on the level of uptake and ongoing use [10,18] by patients who have contracted the SARS-CoV-2 virus and other citizens. An influential modeling study published by a team of researchers at the University of Oxford originally suggested that a 60% adoption rate should be targeted for effective virus transmission reduction, although any level of uptake may help lower disease transmission [19,20]. Even though numerous public consultations have suggested a general willingness to use contact tracing apps during the pandemic [21-28], the available data suggests low rates of continuous use in practice [27,29,30]. For instance, app penetration rates as of March 2021 were as low as 3.6% in France, 6.1% in Japan, 14% in Canada, and 28.5% in the United Kingdom. On the other hand, other countries such as Iceland and Finland have seen higher rates of adoption, at 38.5% and 45.3%, respectively [31,32]. The significant disparity in adoption rates worldwide highlights the fact that certain approaches could be better than others for successful implementation of this emerging technology.

As contact tracing apps encompass many underlying principles and disciplines, multiple aspects can facilitate or hinder their adoption. One of the main caveats in their implementation is concerns over data security and management. Societies are rightfully preoccupied with the challenges in reconciling civil liberties with public health imperatives in a pandemic context [33]. Data privacy, breaches in confidentiality, and the fear of mass surveillance are among the main concerns raised in surveys on user perspectives [21-24,27,28]. Moreover, as with other health informatics interventions, contact tracing apps may generate or exacerbate inequalities if they are not deployed carefully [34,35]. There is a risk of discrimination, repression, and systematic exclusion, especially among communities of color and marginalized groups, which are disproportionately affected by COVID-19 due to structural economic, political, and social vulnerabilities [35-37].

Key factors have been suggested as drivers for widespread success of contact tracing apps: integration with local health policy, adaptable workflows in an ever-evolving context, rapid notification systems, the ability to evaluate the effectiveness of the app transparently, and clear communications addressing privacy concerns [38].

New eHealth initiatives have emerged at an accelerating pace in the last decade; some have seen widespread adoption, whereas others have failed to provide sustained value. These failings can be attributed to design and implementation efforts that were initiated without a good understanding of the interdependencies between technology, societal values, and user experience in a health care setting. Many conceptual frameworks based on implementation science have been developed to evaluate and orient eHealth delivery. These frameworks highlight key factors that predict successful and sustainable eHealth technologies. The urgency of the ongoing public health crisis stimulated the rapid development of contact tracing apps and other eHealth innovations, and this generated a substantial number of related publications. Their coverage of essential design and implementation characteristics for eHealth innovation remains underinvestigated.

### Objectives and Research Questions

The primary objective of this review is to map and analyze the literature on the design and implementation of COVID-19 contact tracing apps. This was achieved through three distinct questions: (1) to what extent does the available literature discuss features that promote the use of contact tracing apps by interested parties? (2) how have patients and citizens been engaged in the design and implementation of these apps? and (3) does the development of these apps correspond to principles of responsible research and innovation?

Through these questions, we studied how the development of contact tracing apps has taken into account considerations related to the uptake and impact of the innovation, the engagement of end users, and the responsible development of eHealth technologies. We ultimately identified the components required for successful and responsible eHealth development as described in the available literature on contact tracing apps and those that are lacking.

## Methods

This study is reported according to PRISMA-ScR (Preferred Reporting Items for Systematic Reviews and Meta-Analyses Extension for Scoping Reviews) guidelines [39] (Multimedia Appendix 1). The scoping review was conducted in accordance with the multistage framework outlined by Arksey and O’Malley [40], as detailed in the following sections.

### Theoretical Frameworks

Three complementary frameworks were selected to address each of the research questions. To answer the first research question on factors that promote uptake of contact tracing apps, we selected the Framework to Improve the Uptake and Impact of eHealth Technologies proposed by van Gemert-Pijnen et al [41]. This framework advocates for a holistic approach comprising six guiding principles and requirements for successful eHealth technology development: (1) participatory processes (eg, upstream involvement of citizens in the selection of app features), (2) continuous evaluation cycles (eg, evaluation of the use and effect of the app on an ongoing basis to update risk assessments), (3) specific actions for implementation (eg, postdesign activities to promote or maintain app uptake), (4) foresight of changes in the organization of health care (eg, interaction of contact tracing apps with traditional public health processes), (5) persuasive design techniques (eg, technology-based suggestions to stimulate the uptake of contact tracing apps), and (6) advanced methods to assess impact (eg, key performance indicators to assess the impact of contact tracing apps). Given the novel application of contact tracing apps in the context of the COVID-19 pandemic, information regarding the latter stages of the framework may understandably be theoretical or perhaps even lacking.

For the second research question, which addresses the engagement of users in the development of contact tracing apps, we selected the Montreal model established by Pomey et al [42], as it focuses on the experiences and knowledge of patients and citizens regarding their health, trajectory of care, and related services [43]. It draws on the Patient and Family Engagement Framework proposed by Carman et al [44] to include a multidimensional assessment of patient engagement in health innovation [45]. For this study, we defined patients as individuals who contracted the SARS-CoV-2 virus. This framework proposes four distinct levels along a patient or citizen engagement continuum, in ascending order of engagement: (1) information (eg, end users have the option of viewing an explanatory video on the proposed app), (2) consultation (eg, a survey to evaluate propensity for app acceptance), (3) collaboration (eg, app adaptation based on recommendations from the public), and (4) coconstruction (eg, governments engage stakeholders to co-design the app). Selected studies that referred to a participatory process were therefore further characterized according to the level of engagement they described.

Finally, for the third research question relating to responsible health innovation, we selected the Responsible Innovation in Health Assessment Tool by Pacifico Silva et al [46]. This framework comes from the field of Responsible Research and Innovation and helps assess health innovations by addressing challenges such as sustainability and equity. The framework elucidates five value domains that need to be considered for responsible innovation: (1) the population health domain, which includes the subvalues of health relevance, ethical, legal and social issues, and health equity (eg, how the app can be made accessible to vulnerable groups); (2) the health system value domain, which includes subsections on inclusiveness, level of care, and responsiveness (eg, contact tracing apps reduce labor from manual contact tracing); (3) the economic domain (eg, cost-effectiveness of contact tracing apps as compared to other public health interventions); (4) the organizational domain (eg, business strategies to increase app value); and (5) the environmental domain (eg, how app architecture can help reduce the carbon footprint).

### Searching for Relevant Studies

A systematic literature search was performed by one author (GG) in five databases (PubMed, Scopus, IEEE Xplore, AMC Digital Library, and Europe PMC) using terms specifically related to the research question. Textbox 1 describes the search strategy used on PubMed. Additional database search strategies are available in Multimedia Appendix 2. The search was first performed on August 26, 2020, and produced 829 results. A second iteration performed on November 6, 2020, generated 1130 results, for a total of 1959 articles.

**Search strategy used on PubMed ([mesh] stands for Medical Subject Headings and indicates that the subject is indexed in the literature; [tw] indicates searches in the title and abstract fields).**

((“contact tracing” [mesh] OR “epidemiological monitoring” [mesh]) AND (“mobile applications” [mesh] OR “algorithms” [mesh] OR “computer security” [mesh] OR “big data” [mesh] OR “computer simulation” [mesh] OR “geographic mapping” [mesh] OR “geographic information systems” [mesh] OR “microcomputers” [mesh] OR “software” [mesh]))

OR (tracing [tw] AND (app [tw] OR apps [tw] OR proximity [tw]))

OR ((contact [tw] OR exposure [tw]) AND notification* [tw] AND (app [tw] OR apps [tw] OR application* [tw]))

OR ((digital* [tw] OR mobile [tw] OR ehealth [tw] OR “eHealth” [tw] OR mhealth [tw] OR “m-health” [tw] OR app [tw] OR apps [tw] OR application* [tw] OR “geolocation*” [tw] OR “location service*” [tw] OR “location system*” [tw] OR “location information” [tw] OR gps [tw] OR big data [tw] OR ((geographic [tw] OR geographical [tw]) AND tracking [tw])) AND (“contact tracing” [tw] OR “contact tracking” [tw] OR “digital epidemiology” [tw]))

### Selecting Studies

The study eligibility criteria (Textbox 2) were informed by a priori knowledge and by the review process itself, in keeping with the scoping review methodology. After removing duplicates, the inclusion and exclusion criteria were applied to the remaining articles.

Each of the 1959 articles was screened by one of two authors (ER and JP), initially excluding those with titles and abstracts unrelated to the topic of study. They then read the remaining articles to determine their relevance to the research question with respect to the inclusion criteria. The senior authors (EO and MPP) screened articles with uncertain relevance for final inclusion or exclusion. Discrepant decisions were resolved through team discussion and consensus. This process resulted in the inclusion of 63 articles, following the search iterations previously described.

**Eligibility criteria.**

**Inclusion criteria**

- Related to design considerations of contact tracing apps for COVID-19 (eg, privacy considerations, citizen inclusion and participatory approach, or incentivization)
- Related to user experience or implementation approaches of contact tracing apps for COVID-19 (eg, surveys or focus groups, strategies to mitigate social vulnerabilities, or recommendations for governments communication with the public)

**Exclusion criteria**

- Published before 2019
- Published in a language other than French or English
- Pertaining exclusively to aspects of computer science or technical developments of contact tracing apps
- Pertaining exclusively to epidemiological feasibility or efficacy of contact tracing apps
- Authors could not obtain access to the full article

### Charting the Data

Data from the included studies was charted on an extraction grid (Multimedia Appendix 3) according to the following categories: authors, title, date of publication, publication stage, aims of the study, and key findings. Articles were also classified into six categories: (1) proposal; (2) comment, editorial, or opinion piece; (3) survey or focus group; (4) case study; (5) review; and (6) essay. A description of each article type is provided in Textbox 3.

**Description of the types of articles.**

**Proposal**

Considers the problems of a particular situation and offers a corresponding solution (eg, proposal to incentivize a contact tracing app [47])

**Comment, editorial, or opinion piece**

Reflects the author’s or journal’s opinion about a subject (eg, contact tracing app effectiveness and data security [48])

**Survey or focus group**

Concentrates on survey or focus group methods for data collection (eg, user acceptability of a contact tracing app [49])

**Case study**

Studies a particular *case* in depth (eg, development of the Trace Together app [50])

**Review**

Examines what has already been discovered about a subject (eg, systematic evaluation of content and features of a contact tracing app [51])

**Essay**

Discusses ideas from the literature in a support of arguments about a specific subject (eg, discussion of an intervention to introduce contact tracing technology [52])

Articles that outlined theoretical recommendations or criticism regarding the overarching concept of contact tracing apps were categorized as being based on theory (eg, ethical considerations of instantaneous contact tracing). In contrast, articles that factually described specific use cases or empirical studies were categorized as being based on practice (eg, description of technical features of a given app and their potential impact on implementation or surveys on user acceptability of a contact tracing app).

Furthermore, the extraction grid included the components of the Gemert-Pijnen et al [41], Pomey et al [42], and Pacifico Silva et al [46] frameworks, as previously discussed. One author (JP) coded each component to determine whether a given article addressed a framework (0 if not present, 1 if present) and extracted the supporting sentences, where applicable. A second author (ER) reviewed each component attribution and supporting sentences, as well as the theory or practice categorization, and reviewed the text for missing information. The principal authors then reviewed the analysis before reporting the results.

### Collating, Summarizing, and Reporting the Results

Figures were produced using codes in accordance with the three theoretical frameworks used and other relevant information such as the date of publication, the type of article, and the theory or practice classification. Visualization of these elements shed light on changes in the available information and on gaps in the underreported domains of design and implementation of contact tracing apps. Critical appraisals of the included articles were beyond the scope of this study.

## Results

**Figure 1:**
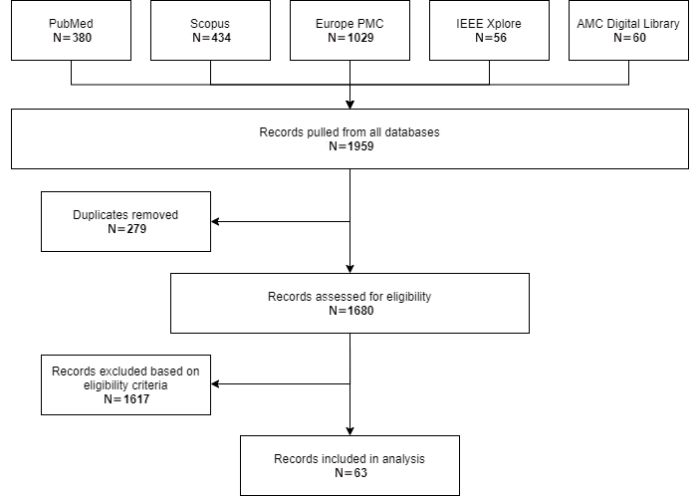
Flowchart of the study selection process.

This scoping review generated a total of 1959 records. Following the removal of duplicates and the application of the selection criteria, 63 articles were included in the analysis (Figure 1).

### Characteristics of Included Studies

**Figure 2:**
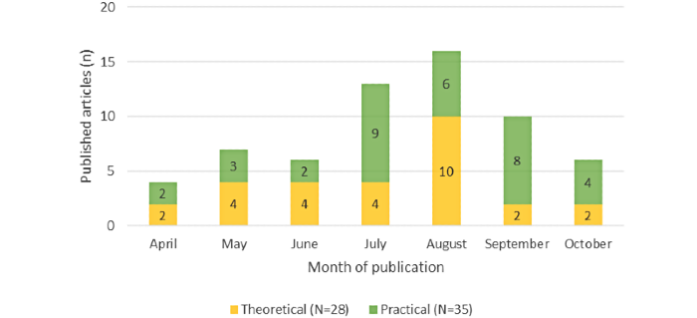
Number of monthly publications/preprints, according to theory- and practice-based categorization.

The included studies (N=63) were published between April 16 and November 6, 2020, in 40 different journals or preprint databases. The majority (n=48, 76%) of included studies were published articles or e-prints, and 24% (n=15) were preprints. The number of published articles peaked in August (n=16, 25%; Figure 2). Theory-based studies were predominant from April to June, whereas the proportion of practice-based articles increased thereafter.

**Figure 3:**
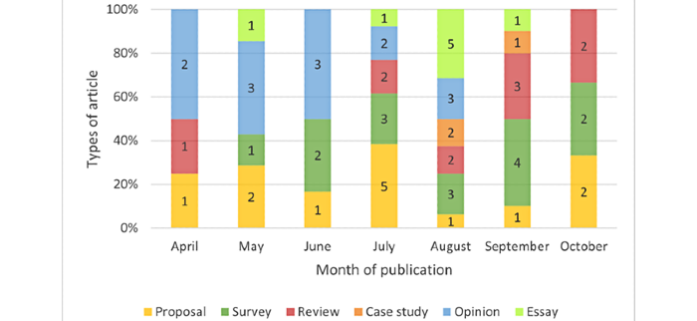
Types of article by month of publication.

Figure 3 illustrates the types of articles by month of publication. Among the 63 included studies, most were surveys (n=16, 25%), followed by proposals and opinion pieces, editorials, or commentaries (n=13, 21%); reviews (n=10, 16%); essays (n=8, 13%); and case studies (n=3, 5%). Opinion pieces, editorials, and commentaries (in blue) were the predominant category before July 2020, followed thereafter by more diversity in publication types. Only surveys (n=1) were illustrated in our analysis in November, but this could be explained by the fact that our second and last iteration was carried out on November 6, 2020.

### Main Findings

#### To What Extent Does the Available Literature Discuss Features That Promote the Use of Contact Tracing Apps by Interested Parties?

**Figure 4:**
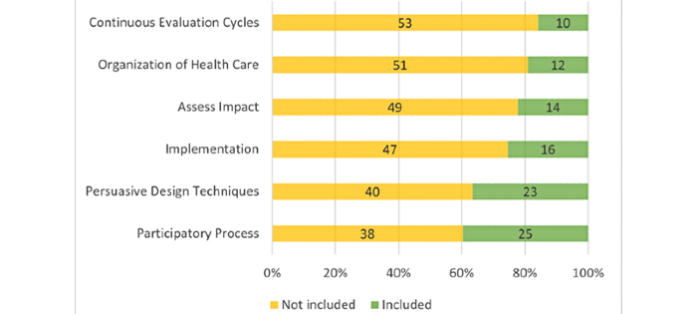
Proportion of articles integrating holistic eHealth development principles.

Overall, all of the six principles of the Framework to Improve the Uptake and Impact of eHealth Technologies [41] were covered in less than half of the selected articles. Among these, mentions of either use cases or recommendations for a participatory process in the development of contact tracing apps appeared in 40% (n=25) of the 63 articles, which made it the most frequently discussed component (Figure 4). Conversely, only 16% (n=10) of the studies discussed continuous evaluation cycles of contact tracing apps. Persuasive design techniques were mentioned in 37% (n=23) of the articles, followed by implementation considerations (n=16, 25%), advanced methods to assess impacts (n=14, 22%), and foresight of changes in the organization of health care (n=12, 19%).

#### How Have Patients and Citizens Been Engaged in the Design and Implementation of These Apps?

**Figure 5:**
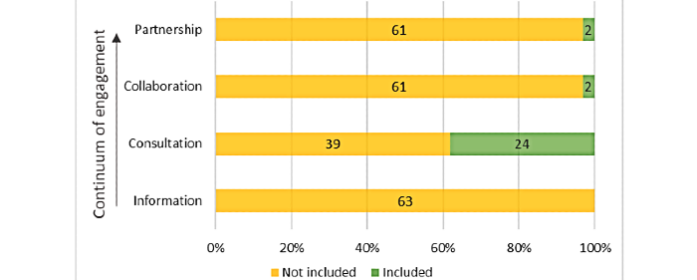
Proportion of articles integrating each level along the patient or citizen engagement continuum.

None of the selected articles referred to documentation given to citizens or patients on COVID-19 contact tracing apps, leaving the Montreal model “information” component [42] unaddressed (Figure 5). On the other hand, stakeholder consultation was the most frequently described level of engagement (n=24, 38%). It is worth noting that only 1 study related to patients and only 1 mentioned the creation of a focus group (in contrast to surveys of the public and reviews of public commentaries on the internet). One article briefly mentioned collaboration, as the responses to the conducted surveys “likely prompted the pivot to wearable tech due to its observation of the public’s reluctance to use their mobile phones for contact-tracing” [50]. Only 3% (2/63) mentioned the importance of partnership in the development and implementation of contact tracing apps, but none of the included articles described an app development process occurring in partnership with citizens or patients.

#### Does the Development of These Apps Correspond to Responsible Research and Innovation?

**Figure 6:**
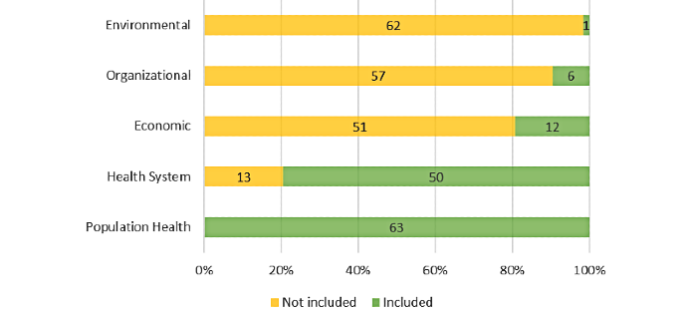
Proportion of articles integrating Responsible Innovation in Health domains.

Among the five domains of the Responsible Innovation in Health Assessment Tool [46], population health appeared in all of the selected studies, constituting the most frequently addressed component (Figure 6). In contrast, only 2% (n=1) of the 63 studies discussed the environmental component. A discussion of the health system was included in 79% (n=50) of the articles, while the economic and organizational dimensions were present in 19% (n=12) and 10% (n=6), respectively.

#### Design Considerations and Implementation Recommendations

The principal considerations and recommendations regarding contact tracing apps in the COVID-19 pandemic context were drawn from the key findings of analyzed articles and are summarized in Table 1. Our results show that the main themes of the 162 theoretical considerations and practical recommendations include data management (n=58, 35.8%), user experience (n=48, 29.6%), communication (n=23, 14.2%), research and implementation methodology (n=18, 11.1%), and the engagement of stakeholders (n=15, 9.3%). Here, user experience refers to the main reactions or perceived barriers that may affect uptake (which mainly comes from survey results), incentivization, app functionality, and other design considerations. Data management designates app architecture, data protection, privacy, monitoring of data, laws governing the use of data, and ethical consideration of data use. Communication covers all recommendations that communication from governments, health authorities, or app developers must be clear and transparent or other measures that may increase public trust in those institutions, such as rebranding and optimizing already existing apps, and reframing the terminology used. The engagement of stakeholders refers to consultation with citizens or patients, strategies to reach vulnerable populations, and theoretical considerations about equity in health access. Finally, research and implementation methodology includes the frameworks proposed to guide future research, general considerations of how to improve uptake, and theoretical considerations of what constitutes ethical app use.

**Table 1:** Themes of considerations and recommendations regarding app design and implementation.

Themes	Considerations and recommendations found in the literature (N=162), n (%)
Data management (eg, the use of blockchain [53-56] and sunset clause [57])	58 (35.8)
User experience (eg, rewards to app users [58] and simple design and user interface for interaction [59])	48 (29.6)
Communication (eg, clarifying false beliefs about the app [29] and prevention campaign about individual risks [60])	23 (14.2)
Research and implementation methodology (eg, NPT^a^ framework to guide development and evaluation of complex DPT^b^ interventions [61], apps must be necessary, proportional, scientifically valid, and time bound [62])	18 (11.1)
Engagement of stakeholders (eg, low-cost wristband in low-socioeconomic areas [54] or alignment of the app with local culture and vulnerable populations [63])	15 (9.3)

^a^NPT: Normalization Process Theory.

^b^DPT: digital proximity tracing.

## Discussion

### Principal Findings

This study demonstrates that the volume of articles on design and implementation considerations for COVID-19 contact tracing apps grew rapidly in the first months of the pandemic and peaked in August 2020. As the evidence base increased, the proportion of opinion pieces decreased in comparison to surveys and review articles. This is not surprising given the novelty of this technology and the urgency around technological developments triggered by the pandemic. Accordingly, earlier articles mostly discussed principles related to the development and implementation of contact tracing apps mostly in theoretical terms, while the proportion of empirical articles increased starting in July. Despite the growing number of publications, their scope remained limited with respect to design and implementation considerations. Our findings demonstrate that critical design and implementation considerations were lacking in the early academic literature on contact tracing apps.

In fact, less than half of the selected articles cited the need for a participatory process, which nonetheless constituted the most frequently referenced item of the Framework to Improve the Uptake and Impact of eHealth Technologies. Only 40% (25/63) of the articles presented evidence of public or patient engagement in the contact tracing app development process. This stands in stark contrast with the ideal of “co-creation from ideation to operationalization” in eHealth technologies, as described in this framework [41]. Other principles from the same framework were infrequently addressed, such as the need for continuous evaluation cycles, the creation of new processes for health care delivery, the need to assess impact, and the proposing of specific actions for implementation.

We further analyzed the level of citizen and patient engagement in the development of contact tracing apps by using the Montreal model [42]. We found that, among the 28 studies that addressed this component, nearly all (n=24, 86%) consisted of public consultations. Although essential to inform design and implementation of eHealth technologies, consultation ranks lower on the patient engagement continuum as compared to collaboration and partnership. This imbalance may have resulted from the urgency of the public health crisis combined with the perception that citizen and patient engagement is time-consuming [64-66]. However, the upstream efforts deployed to promote collaboration and partnership with users provided key insights at early stages of development [67,68]. Ignoring user engagement or relying solely on downstream consultation may in fact delay implementation by triggering a need to redesign certain features or even contribute to ultimately unsuccessful ventures [68,69].

Moreover, when we assessed these studies according to the Responsible Innovation in Health framework [46], we found that most of the 63 articles described the impact of contact tracing apps at the population level and on health systems (n=63, 100% and n=50, 79%, respectively), mainly in theoretical terms. However, only a few acknowledged the environmental, organizational, and economic value domains (each domain was found in less than 20% of the included studies). Their near absence from the literature may indicate that these domains are not perceived as being relevant to the development of COVID-19 contact tracing apps.

The incompleteness of the academic literature on the design and implementation characteristics of COVID-19 contact tracing apps stems in part from the novelty of the topic and the need for timely innovations to fight the pandemic. As such, we did not expect the literature to address all the domains of the selected frameworks. However, few academic publications highlighted the need to assess the impact of this intervention or consider organizational challenges related to its deployment, among other key elements to successful and responsible eHealth innovation. Likewise, the limited references to higher forms of upstream end user engagement stand in contrast to this technology’s inherent reliance on widespread adoption. It may be that app developers or researchers did not perceive some of these factors as bearing sufficient relevance for incorporation at the development stage or for later publication. Nonetheless, more than one year since the start of the pandemic, many of these elements are still lacking and were highlighted as essential to the success of COVID-19 contact tracing apps [38]. Considering these findings, we hypothesize that the sense of urgency instilled by the pandemic motivated shortcuts away from a full compliance with best practices in eHealth development. In turn, this may have contributed to the unconvincing implementation of contact tracing apps to date. Future research will help determine which factors are most associated with the development of sustainable, feasible, ethically acceptable, and socially desirable technological solutions in the context of a public health emergency. As specific evidence on this topic continues to accumulate, we draw upon the existing literature to make some practical recommendations for successful contact tracing apps and related eHealth innovations.

### Recommendations Regarding the Design Considerations and Implementation of COVID-19 Contact Tracing Apps

#### Data Management

Data protection and the looming risk of mass surveillance have understandably dominated the debate on the ethical, legal, and social implications of contact tracing apps. Users must therefore be intelligibly informed of the steps taken to protect their privacy. One consideration is that the risk of the proposed contact tracing app should be compared with that of the frequently used apps that most people have on their cellphones. For example, the New York Times “Privacy Project” recently revealed the tracking of millions of unsuspecting Americans through location-sharing apps (eg, apps used to access directions, weather information, or local fidelity programs) [70]. The heightened public scrutiny of contact tracing apps may thus serve as an opportunity to promote digital literacy and reflect on the responsible use (or misuse) of digital tools.

#### Communication

Clear and transparent messaging developed with and for citizens can be promoted through simple and familiar means, such as concise information labels inspired by those of the food industry. This approach was used by members of the health care machine learning community to promote the transparent and responsible use of clinical decision support tools [71]. If they are shown to be effective, clear communication and efforts to improve health literacy will further promote stakeholders’ engagement [72,73] and may increase public trust in governments and health authorities. Rebranding or reframing such as changing the terminology from tracking apps to exposure notification apps may also be a solution that would improve both public trust and app uptake. Moreover, given the large number of contact tracing apps developed thus far, a consistent presentation of fundamental design components (see the Introduction section) would assist stakeholders in their evaluation of a given app. It may also promote efficient comparisons between different apps and perhaps limit the risk of unnecessary duplication.

#### Engaging Key Stakeholders

Key stakeholders of contact tracing apps include potential users, technology developers, policy makers, and funding agencies. As previously noted, the prevailing form of engagement has been through public consultations, which is recommended in the analyzed articles. We would therefore argue that app developers and the various institutions that implement such technologies would benefit from greater upstream collaboration and partnership with individuals from diverse backgrounds, including patients. Engaging key stakeholders early in the process will also help identify the right problem and constraints, eventually narrowing the range of most suitable technological tools. Indeed, even if a contact tracing app perfectly identified high-risk contacts, it would not achieve the desired outcome of reduced viral transmission if it required prohibitively expensive hardware or if it relied on massive viral testing in a strained health care system unable to provide a sufficient number of diagnostic tests.

As previously described, the potential risks of contact tracing apps may disproportionately affect minority and marginalized groups [35-37]. It is important to seek such participation in the development of these tools to mitigate and, ideally, eliminate the risk of increasing disparities through their use. This recommendation is also supported by the literature, which prescribes strategies for reaching low socioeconomic groups and older adults. Unfortunately, it may be difficult to engage with certain groups, such as for people who experience homelessness, (digital) illiteracy, living in remote settings, or underrepresentation within the typical channels of citizen engagement due to systemic racism or other structural vulnerabilities. In these cases, one practical option by which app developers could enhance the diversity of voices contributing to their product is to partner with community leaders and individuals who work closely with underrepresented groups. Although this will require an early investment of time and resources, the dividends in terms of greater inclusiveness, accurate problem identification, and a broader assessment of the impact on outcomes across different populations will strengthen a project’s chances of achieving responsible design and successful implementation. Public and private funders can also play an important role in ensuring that effective collaboration occurs early in the development of contact tracing apps by prioritizing proposals that promote a participatory process.

#### User Experience

A simple and intuitive user interface may not only enhance the user experience at an individual level, it may also improve uptake at the population level. Apps that focus on interactive design features can help users better understand how to use the app correctly and more effectively, whereas apps that are focused on information may reduce the assimilation of this information by users if they are not visually appealing [74]. Moreover, resolving in-app technical shortcomings will likely improve the user experience. In fact, an Australian survey noted that 24% of respondents had listed technical concerns as a reason not to download a contact tracing app during the COVID-19 pandemic [27]. An easy system for reporting technical difficulties and a minimally disruptive evaluation of other app functionalities may therefore increase use. Moreover, since one main barrier to app uptake is privacy and data security concerns [21-23,27,29,49,75,76], users can be empowered by designs that use customizable app functionalities. For example, app users could be offered choices in the technology used to collect the data (GPS or Bluetooth), how the data is stored [77], and the level of interaction with local health agencies.

Simple and useful apps will likely incentivize uptake and use. This can be supported by developing apps that require readily available personal technology, personalized updates on pertinent and accurate information aligned with local guidelines, minimal disruption to daily functioning (eg, minimizing battery use [78]), and facilitated testing in collaboration with local health care systems. Poor coordination with local health actors may therefore significantly compromise the potential user value of contact tracing apps. On the other hand, locally integrated apps may help streamline testing when it is indicated and provide current trustworthy recommendations. In many instances, COVID-19 contact tracing apps were designed to complement established traditional public health interventions. Apps that work in silos may therefore be less appealing to the public, as their perceived usefulness and effectiveness may be compromised [79]. Furthermore, implementation should aim for interoperability in the identification and notification of high-risk contacts in the greater interest of public health. This may be more attainable in jurisdictions that share compatible legal frameworks for data protection and privacy, such as the General Data Protection Regulation [80].

Financial incentives should also be considered. A study that focused on tracking the use of Germany’s official contact tracing app, Corona-Warn-App, found that app uptake is more prevalent among older populations, individuals with pre-existing conditions, and those with high levels of education and income. Additionally, the study reported that information interventions, in the form of short videos addressing privacy, effectiveness, and app functionality issues, were useful in increasing users’ knowledge about the app but were not effective in driving uptake. On the other hand, interventions that provide a monetary incentive (as low as €1 [US $1.22], €3 [US $3.67], or €5 [US $6.11]) upon installation were found to be useful in increasing uptake [81].

#### Research and Implementation Strategies

Empirical evidence is urgently needed to determine whether the benefits of a given contact tracing app significantly outweigh its risks. Although contact tracing technologies were used in prior Ebola and influenza outbreaks, there is limited empirical evidence on their effectiveness. As for their impact in reducing viral transmission during the COVID-19 pandemic, it is mainly based on mathematical simulation models that used varied assumptions and methodologies [18]. Real-life estimated treatment effects, beyond the current simulation models, are urgently needed, with precise descriptions of the context in which the tool was used. In addition to the estimated effect of contact tracing apps on public health outcomes (eg, the impact on the basic reproduction number R_0_), studies should also report on the input data and the corresponding outputs. At the least, information on app downloading and daily use must be made available, along with a description of key app features. Such information would enable the study of factors that enhance uptake and the relationship between app uptake and public health effectiveness. Observational studies on contact tracing app effectiveness during a period when large-scale interventions are continuously being proposed and deimplemented will certainly be limited by significant biases. They can nonetheless shed light on the role played by digital contact tracing during this pandemic and future infectious disease outbreaks. This observational evidence can then be compared to outcomes from simulation-based studies, including a recent modeling that suggests that a digital contact tracing and exposure notification system can support traditional public health interventions in reducing transmission, even at participation levels as low as 15% [82].

In addition, academic reports on contact tracing apps and related eHealth innovations must adhere to established reporting guidelines such as CONSORT-EHEALTH (Consolidated Standards of Reporting Trials of Electronic and Mobile Health Applications and Online Telehealth) for eHealth and mHealth interventions [83]. The global scale of contact tracing apps makes the incorporation of the key domains and subdomains proposed in the Responsible Innovation in Health framework even more crucial than in local innovations. eHealth proposals should therefore indicate their predicted or estimated impact on health inequalities, health system inclusiveness and responsiveness, frugality, business model, and eco-responsibility. Funding agencies and academic journals must request that submissions comprehensively address these issues.

### Limitations

The findings of this scoping review must be interpreted considering certain limitations. First, this study includes articles published up to November 6, 2020. As the volume of publications continues to grow, a systematic review focused on narrower questions related to contact tracing apps may become relevant. We attempted to maximize the reach of our search by including multiple databases and by developing a rigorous study selection process. Relevant articles only available in the gray literature or exclusively in governmental databases may nevertheless have been missed, although official apps developed by governments, such as the National Health Service contact tracing app, were included in several of the articles analyzed. Furthermore, given the purposefully broad question posed by this study, the reproducibility of data extraction and charting presented some challenges. We addressed them by relying on well-established and complementary frameworks that were particularly appropriate to the research question. Moreover, multiple authors reviewed the process and provided supportive statements when a particular component or domain was considered to be present in a given article.

### Conclusions

The emerging academic literature on contact tracing apps reveals significant knowledge gaps regarding their design and implementation. Key stakeholders are thus limited in their ability to critically appraise this eHealth innovation. Most of the included studies lacked fundamental aspects of the successful eHealth development and implementation framework. Similarly, few articles described the impact of contact tracing apps on the environmental, organizational, and economic domains, which are essential to evaluate responsible innovation in health. Among the studies that described a form of public participation, nearly all of them relied on consultation as opposed to collaboration or partnership. These overlooked components of eHealth development and implementation may have contributed to the modest uptake of contact tracing tools worldwide. They suggest a critical gap between theory and practice, whereby numerous academic sources promote a holistic and participatory approach to eHealth innovation, but few products incorporate them. Partnerships between app developers, researchers, policy makers, and users early in the development process will narrow this gap. Transparent, systematic, and comprehensive reporting of COVID-19 contact tracing app outcomes will further enable their critical appraisal. The lessons learned about the social acceptability of contact tracing apps as they were deployed at an unprecedented pace and scale must serve in future iterations of this innovation and in the development of other eHealth technologies aimed at sustainably supporting public health. They must attest to the importance of stakeholder engagement, problem identification, minimal system disruptions, longitudinal outcome measurement, and use incentivization.

## Appendix

Multimedia Appendix 1 PRISMA-ScR (Preferred Reporting Items for Systematic Reviews and Meta-Analyses Extension for Scoping Reviews) Checklist.

Multimedia Appendix 2 Additional database search strategies.

Multimedia Appendix 3 Database of included articles and analysis.

## Abbreviations

CONSORT-EHEALTH: Consolidated Standards of Reporting Trials of Electronic and Mobile Health Applications and Online Telehealth
PRISMA-ScR: Preferred Reporting Items for Systematic Reviews and Meta-Analyses Extension for Scoping Reviews
