# Supplementary material for: Considerations for the Design and Implementation of COVID-19 Contact Tracing Apps: Scoping Review
Source: JMIR Mhealth Uhealth. 2021 Jun 9;9(6):e27102. doi: 10.2196/27102 (PMC8191727; doi:10.2196/27102)
Supplement: Multimedia Appendix 2 [file mhealth_v9i6e27102_app2.pdf]

Search strategies used for research in different databases

Scopus

(
TITLE-ABS-KEY(tracing AND (app OR apps OR proximity))

OR TITLE-ABS-KEY((contact OR exposure) AND “notification*” AND (app OR apps OR application*))

OR TITLE-ABS-KEY((digital* OR mobile OR ehealth OR "e-health" OR mhealth OR "m-health" OR app OR apps OR application* OR geolocation* OR "location service*" OR "location system*" OR "location information" OR gps OR “big data” OR ((geographic OR geographical) AND tracking)) AND ("contact tracing" OR "contact tracking" OR "digital epidemiology"))
)

IEEE Xplore

(
("All Metadata":"tracing" AND ("All Metadata":app OR apps OR proximity))

OR (("All Metadata":contact OR exposure) AND ("All Metadata":notification*) AND ("All Metadata":app OR apps OR application*))

OR (("All Metadata":digital* OR mobile OR ehealth OR "e-health" OR mhealth OR "m-health" OR app OR apps OR application* OR geolocation OR "location service*" OR "location system*" OR "location information" OR gps OR “big data” OR ((geographic OR geographical) AND tracking)) AND ("All Metadata":"contact tracing" OR "contact tracking" OR "digital epidemiology"))
)

AMC Digital Library:

Title:(tracing AND (app OR apps OR proximity)) OR Abstract:(tracing AND (app OR apps OR proximity)) OR Title:(((contact OR exposure) AND “notification*” AND (app OR apps OR application*))) OR Abstract:((contact OR exposure) AND “notification*” AND (app OR apps OR application*)) OR Title:((digital* OR mobile OR ehealth OR "e-health" OR mhealth OR "m-health" OR app OR apps OR application* OR geolocation* OR "location service*" OR "location system*" OR "location information" OR gps OR “big data” OR ((geographic OR geographical) AND tracking)) AND ("contact tracing" OR "contact tracking" OR "digital epidemiology")) OR Abstract:((digital* OR mobile OR ehealth OR "e-health" OR mhealth OR "m-health" OR app OR apps OR application* OR geolocation* OR "location service*" OR "location system*" OR "location information" OR gps OR “big data” OR ((geographic OR geographical) AND tracking)) AND ("contact tracing" OR "contact tracking" OR "digital epidemiology"))

Executed as:
[[Publication Title: tracing] AND [[Publication Title: app] OR [Publication Title: apps] OR [Publication Title: proximity]]] OR [[Abstract: tracing] AND [[Abstract: app] OR

[Abstract: apps] OR [Abstract: proximity]]] OR [[[Publication Title: contact] OR [Publication Title: exposure]] AND [Publication Title: "notification*"] AND [[Publication Title: app] OR [Publication Title: apps] OR [Publication Title: application*]]] OR [[[Abstract: contact] OR [Abstract: exposure]] AND [Abstract: "notification*"] AND [[Abstract: app] OR [Abstract: apps] OR [Abstract: application*]]] OR [[[Publication Title: digital*] OR [Publication Title: mobile] OR [Publication Title: ehealth] OR [Publication Title: "e-health"] OR [Publication Title: mhealth] OR [Publication Title: "m-health"] OR [Publication Title: app] OR [Publication Title: apps] OR [Publication Title: application*] OR [Publication Title: geolocation*] OR [Publication Title: "location service*"] OR [Publication Title: "location system*"] OR [Publication Title: "location information"] OR [Publication Title: gps] OR [Publication Title: "big data"] OR [[[Publication Title: geographic] OR [Publication Title: geographical]] AND [Publication Title: tracking]]] AND [[Publication Title: "contact tracing"] OR [Publication Title: "contact tracking"] OR [Publication Title: "digital epidemiology"]]] OR [[[Abstract: digital*] OR [Abstract: mobile] OR [Abstract: ehealth] OR [Abstract: "e-health"] OR [Abstract: mhealth] OR [Abstract: "m-health"] OR [Abstract: app] OR [Abstract: apps] OR [Abstract: application*] OR [Abstract: geolocation*] OR [Abstract: "location service*"] OR [Abstract: "location system*"] OR [Abstract: "location information"] OR [Abstract: gps] OR [Abstract: "big data"] OR [[[Abstract: geographic] OR [Abstract: geographical]] AND [Abstract: tracking]]] AND [[Abstract: "contact tracing"] OR [Abstract: "contact tracking"] OR [Abstract: "digital epidemiology"]]] AND [Publication Date: (01/01/2019 TO 12/31/2020)]

<u>Europe PMC:</u>

(
(tracing AND (app OR apps OR proximity))

OR ((contact OR exposure) AND “notification*” AND (app OR apps OR application*))

OR ((digital* OR mobile OR ehealth OR "e-health" OR mhealth OR "m-health" OR app OR apps OR application* OR geolocation* OR "location service*" OR "location system*" OR "location information" OR gps OR “big data” OR ((geographic OR geographical) AND tracking)) AND ("contact tracing" OR "contact tracking" OR "digital epidemiology"))
)

AND (SRC:PPR)
